# Performance of behavioral assays: the Rat Grimace Scale, burrowing activity and a composite behavior score to identify visceral pain in an acute and chronic colitis model

**DOI:** 10.1097/PR9.0000000000000712

**Published:** 2019-03-05

**Authors:** Vivian S.Y. Leung, Marie-Odile Benoit-Biancamano, Daniel S.J. Pang

**Affiliations:** Faculty of Veterinary Medicine, Université de Montréal, Saint-Hyacinthe, QC, Canada

**Keywords:** Ongoing pain, Pain assessment, Animal, Rodent, Pain behavior, Facial expression, Spontaneous pain

## Abstract

**Introduction:**

The Rat Grimace Scale (RGS), a facial expression scale, quantifies the affective component of pain in rats. The RGS was developed to identify acute and inflammatory pain, and applicability in acute and chronic visceral pain is unknown. The dextran sulfate sodium (DSS) colitis model is commonly used in rats, but pain is rarely assessed, instead, disease progression is monitored with the Disease Activity Index (DAI; assessing fecal blood, stool consistency, and weight loss).

**Objectives:**

The aim of this study was to assess whether the RGS and 2 additional behavioral tools (composite behavior score [CBS] and burrowing) could identify pain in an acute and chronic DSS colitis model.

**Methods:**

Male and female Sprague-Dawley rats were block randomized to (1) acute colitis (4 days DSS in drinking water); (2) chronic colitis (4 days DSS, 7 days water, and 3 days DSS); or (3) control (14 days water). Disease Activity Index, RGS, CBS, and burrowing assessments were performed daily.

**Results:**

Rat Grimace Scale scores increased as DAI scores increased during both acute and chronic phases. Burrowing only decreased during the acute phase. By contrast, CBS scores did not increase significantly during either colitis phase.

**Conclusions:**

These data show that the RGS and burrowing did not decrease in a sustained manner during chronic phase visceral pain, and that variables assessed in the DAI are indicative of pain. This suggests that the RGS can be applied to a wider range of pain types and chronicity than originally suggested. These findings increase the application of the RGS as a pain scale and welfare improvement tool.

## Abstract 2

Supplemental Digital Content is Available in the Text.

The ability of spontaneous behavioral assays to assess chronic pain is limited. In colitis, RGS identified acute and chronic pain, burrowing identified acute pain.

## 1. Introduction

In recent years, it has been proposed that spontaneous behaviors of animals should be used to assess pain in animals.^22,25^ In laboratory rats, one of these behavioral tools is the Rat Grimace Scale (RGS), a facial expression scale, which was developed with acute inflammatory pain models.^28^ Since its initial development, performance of the RGS in acute inflammatory pain models has been confirmed,^7,18^ and its application in other acute and neuropathic pain models has been described.^1,19^ Development of the Mouse Grimace Scale identified a limited ability of this scale to identify pain in classic models of neuropathic pain (chronic constriction injury and spared nerve injury), but there has been little investigation of chronic pain using other grimace scales, including the RGS.^1,16^ Furthermore, a study of induced acute visceral mucositis failed to identify significant changes in the RGS.^33^ Therefore, it is currently unclear what role the RGS may play in the evaluation of chronic or visceral pain. Potential alternative, or complementary, methods to the RGS include a composite behavior score (CBS) and burrowing behavior.^2,26^ The CBS, which uses an ethogram, including twitching, writhing, and back-arching behaviors, has been used successfully to assess visceral pain in laparotomy and mucositis models.^26,33^ Burrowing, as an expression of voluntary behavior, is performed by a high proportion of laboratory rats.^2^ It has been successfully applied in models of induced osteoarthritis and found to be robust in multicenter testing.^34^

The dextran sulfate sodium (DSS) colitis model is well characterized and widely used to study colitis in mice and rats.^6,9,23^ With a focus on underlying disease mechanisms, the assessment of pain is performed infrequently in this model despite being a common symptom of clinical disease.^8^ Where pain is evaluated, it is typically limited to nonspecific behaviors or evoked hypersensitivity testing,^13,17,20,30,31^ measures that may not capture the pain experience.^21,27^ Model severity and progression are commonly monitored using the Disease Activity Index (DAI), which scores the presence of fecal blood, stool consistency, and weight loss.^6^ A relationship between similar clinical signs and pain is present in people but has not been established in rodent models of colitis.^3,4^

The aim of this study was to assess the performance of the RGS, CBS, and burrowing as measures of acute and chronic visceral pain in a DSS-colitis rat model. We hypothesized that the RGS and CBS would increase in parallel with the DAI, with a concurrent reduction in burrowing.

## 2. Methods

### 2.1. Ethical statement

All experiments were approved by the institutional animal care and use committee (Comité d'Éthique de l'Utilisation des Animaux of Université de Montréal, #Rech-1876) and performed in accordance with the Canadian Council on Animal Care guidelines.

### 2.2. Animals

Thirty-eight male and female Sprague-Dawley rats of at least 6 weeks of age (females [n = 18]: 182 g [range: 144–289 g]; males (n = 20): 217 g [range: 183 293 g]) were obtained from Charles River Laboratories (Sherbrooke, Canada). Animals were housed singly in polycarbonate rat cages (2154F, Tecniplast, Montreal, QC, Canada) in a conventional facility. Single housing was required to facilitate daily DAI assessments (stool consistency and presence of blood). Rats had hardwood laboratory bedding (Beta Chip, Charles River Laboratories, Sherbrooke, Canada), with a plastic tube (ABS tubing, Verdun, IPEX Inc, QC, Canada) and a nylon toy for enrichment (Bio-serv Inc, Flemington, NJ). They were housed in a 14:10-hour light/dark cycle with lights on at 6 am and temperature and humidity settings of 22°C and 35% to 50%, respectively. Rats were fed laboratory rat pellets (Charles River Rodent Diet #5075, Charles River Laboratories, Sherbrooke, QC, Canada), and tap water was provided ad libitum before the start of the study. Rats acclimatized to their new surroundings for at least 3 days before habituation procedures began.

### 2.3. Colitis model induction

**Figure 1.**
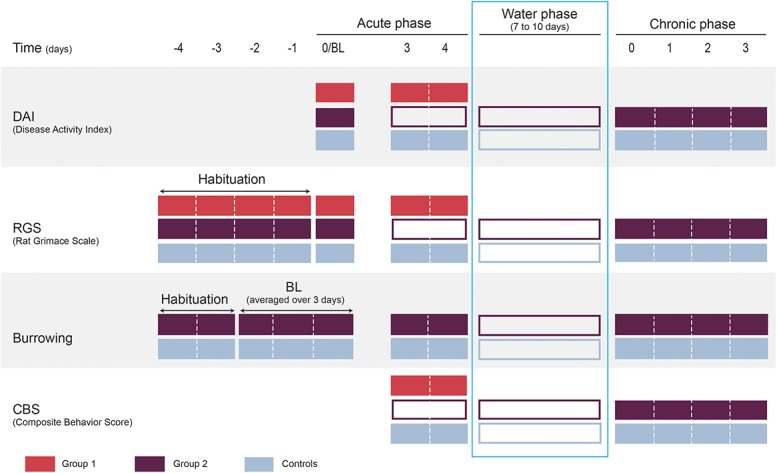
Experimental timeline (DAI, RGS, burrowing, and CBS). Each filled box indicates a habituation or assessment activity for each assessment method. Unfilled boxes indicate when no assessments were performed. During the acute phase, group 1 and group 2 rats were treated with 5% DSS administered in water. Group 1 rats were euthanized at the end of the acute colitis phase. During the water phase (group 2 and controls), no assessments were made. During the chronic phase, group 2 rats were treated with 5% DSS for a second time before euthanasia on day 3. Tissue was harvested for microscopic and macroscopic analysis immediately after euthanasia. BL, baseline; DSS, dextran sulfate sodium.

Colitis was induced by adding DSS (5%, J63606, Alfa Aesar, Ward Hill, MA, MW 40,000) in to distilled drinking water provided ad libitum. The DSS solution was prepared on the day of administration (day 0). Rats were block randomized with a list randomizer (random.org) with equal allocation of sexes to 1 of 3 treatment groups: (1) group 1 (n = 12) was given one phase of DSS (acute phase); (2) group 2 (n = 13) was given one phase of DSS (acute phase) followed by a water phase (distilled drinking water only), then a second phase of DSS (chronic phase); and (3) controls (n = 13) were given distilled drinking water for the duration of the experiment (Fig. 1). Randomization was performed after baseline (BL) assessments. Dextran sulfate sodium treatments were stopped when all rats within each block-randomized cohort displayed signs of colitis as indicated by the DAI (ie, decrease in stool consistency, bloody stools, and weight loss), with an average DAI score of 2/4. The water phase was terminated when all rats within the cohort had DAI scores of 0 for at least 24 hours before restarting DSS treatment. After completion of the final assessments, rats were euthanized (induction of general anesthesia with isoflurane, followed by guillotine decapitation after confirming loss of righting and pedal withdrawal reflexes): on day 4 of the acute phase for group 1, on day 3 of the chronic phase for group 2, and the equivalent day for group 3. All assessments (DAI, RGS, CBS, and burrowing) were performed during the light phase. Disease Activity Index and RGS were assessed in a room adjacent to the housing room. Burrowing was assessed in the housing room.

### 2.4. Habituation

Before the study, all rats were habituated to the observer (V.L., Fig. 1). On the day before habituation (day −5), 2 pieces of food reward (Honey Net Cheerios, General Mills, Inc, Golden Valley, MN) were introduced to each cage. For 4 days (day −4 to −1), rats were handled by the experimenter for a minimum of 10 minutes each while offering the food reward. Rats were also habituated to the Plexiglas observation box (28 cm length × 15 cm width × 21 cm height) daily, whereby they were placed inside for a maximum of 10 minutes with a food reward.

### 2.5. Disease Activity Index

The DAI consists of 3 items, each scored from 0 to 4: weight loss, stool consistency, and bloody stools (Table 1).^6^ Rats were weighed after completion of all assessments (RGS, CBS, and burrowing). If gross bleeding was not evident, the presence of blood was assessed with a fecal blood slide test (Hemoccult II Slides, 60151A, Beckman Coulter, Inc, Brea, CA).

**Table 1:**
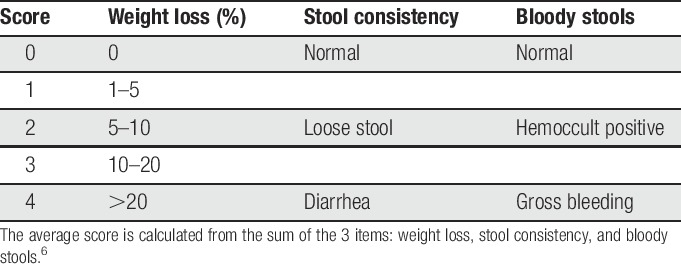
Disease Activity Index scoring.

### 2.6. Rat Grimace Scale

The RGS was scored as originally described by Sotocinal et al.^28^ Briefly, each of 4 action units (orbital tightening, nose/cheek flattening, ear changes, and whisker changes) was assigned a score of 0, 1, or 2 based on degree of presentation.

The RGS was scored in 2 ways: (1) in real time and (2) with video-based analysis. During real-time scoring, observations began 3 minutes after introducing the rat to the observation chamber. Facial expression was scored based on a 15-second observation period repeated every 30 seconds, which generated a total of 18 scores for each time point over a 9-minute time period.^18^ Scores were averaged every 3-minute interval, and the resultant 3 scores were averaged again for a final score. For video-based analysis, video recording took place at the same time as real-time observations. Blinded video-based scoring was performed in “real-time” while the video was playing to assess observer bias because it was not possible to blind the observer from treatment groups and time points.^18^ Video-based data were used for analysis. Both real-time and video scores were performed by the same observer (V.L.). The observer was previously trained in RGS scoring by an experienced rater.^35^ Real-time scoring was performed between 8 am and 12 pm, and the order in which the rats were assessed was randomized each day with a list randomizer (random.org).

### 2.7. Burrowing

The technique described by Andrews et al.^2^ was followed. During 2 days of habituation (days −4 to −3; Fig. 1), rats were placed in same sex pairs in a 53-L box (burrowing box; 58.4 cm length × 41.3 cm width × 31.4 cm height; Sterilite Corporation, Townsend, MA) with the empty burrowing tube (32 cm in length × 10 cm in diameter, elevated by 6 cm at the open end of the tube with 2 metal legs) for 30 minutes. After 30 minutes, the burrowing tube was filled with 2.5 kg of gravel (2–5 mm, Premium Aquarium Gravel, Clifford W. Estes Company, Fairfield, NJ) and placed with the rats for 60 minutes. If a pair of rats did not burrow sufficiently (<100 g of gravel displaced) on the first day of habituation (day −4), a new pair was created including a burrowing rat (identified on day −4) and the protocol repeated. Baseline assessments were made over the next 3 days (days −2 to 0) with rats placed individually in the burrowing box with the gravel-filled burrowing tube for 60 minutes daily. The amounts of gravel displaced over these 3 days were averaged to produce a BL score for each rat. It was predetermined that rats that had a BL of less than 100 g of gravel displaced would be excluded. Burrowing assessments were always performed after RGS scoring. Burrowing was assessed in group 2 and control animals during both acute and chronic phases.

### 2.8. Composite behavior score

The CBS consisted of recording the frequency of 5 behaviours (writhing, vertical back arching, stagger/fall, twitch, and belly pressing) as described by Roughan and Flecknell^26^ and Thomas et al.^29^ Writhing behavior was defined as the contraction of the abdominal muscles. Back arching was defined as a vertical stretch upward that resembled a cat stretching. Stagger/fall behavior was defined as a rat falling over or losing its balance while moving. Twitch behavior was defined as a fleeting contraction of flank muscles. These behaviours were observed from the same video recordings used for the RGS (observer blinded to treatment). The total frequency of each behavior was summed to produce a total score.

### 2.9. Macroscopic scores

After euthanasia, abdomens were opened through a midline incision and colons removed. Macroscopic scoring consisted of body weight loss from BL, changes in colon length compared with controls, adhesion of the colon to the mesentery, length of any ulcer present, percentage of colon inflamed, presence of erythema, fecal blood, diarrhea, and bowel thickness.^5^ Ulcer length and bowel thickness were measured with digital calipers after fixation in formalin for 48 hours. The score for each item was summed to provide a total macroscopic score (Table 2).

**Table 2:**
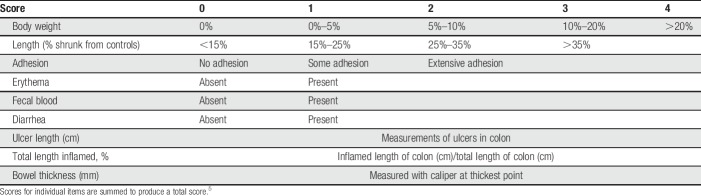
Macroscopic scoring of colon samples.

### 2.10. Microscopic

Colons were collected and fixed in neutral buffered 10% formalin for approximately 48 hours before 4 samples (7-mm transverse sections) were collected from the distal colon of each rat. Any ulcers identified were transected, and both halves examined. Tissues were routinely processed, and slides were cut at 4 um and stained with hematoxylin–eosin–phloxine–saffron. The microscopic assessments consisted of 3 items (severity of inflammation, mucosal damage, and crypt damage) and the highest score used for analysis (Table 3).^32^ Each item was then multiplied by the factor of the pathological change rate, taking into account the total surface of the affected area.

**Table 3:**
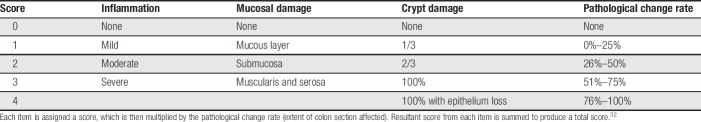
Microscopic scoring of colon samples.

### 2.11. Humane endpoints

Humane endpoints were assessed daily and consisted of (1) more than 15% weight loss, (2) an RGS score of 2/2 for more than 4 hours, (3) a DAI score ≥3/4, and (4) obvious lethargy. Any rat that reached an endpoint was euthanized.

### 2.12. Statistical analysis

Data were analyzed and sample size estimated with commercial statistical software (Prism 6.07, GraphPad Software, La Jolla, CA; MedCalc Software 18.5, Ostend, Belgium and G*Power 3.1.9.2, Germany). All data, except the CBS and pathology data (macroscopic and microscopic scores), approximated a normal distribution according to the D'Agostino–Pearson omnibus normality test. Comparisons between DSS-treated groups and controls were performed with a 2-way analysis of variance followed by a post hoc Bonferroni test. Comparisons within groups (from BL) were performed with a 2-way analysis of variance followed by a post hoc Dunnett test (RGS, CBS, DAI, and burrowing) and a Kruskall–Wallis test for microscopic and macroscopic scores (Dunn's post hoc test). A Bland–Altman analysis of repeated measures was used to assess whether RGS real-time and video scores were similar. Sample sizes were estimated for the primary outcomes of interest; the RGS, CBS, and burrowing. For the RGS: a sample size of 12 animals per group was estimated based on an alpha of 0.05, power of 0.8, SD of 0.25, and a mean difference of 0.3.^18^ For the DAI: a sample size of 12 animals per group was estimated based on an alpha of 0.05, beta of 0.8, SD of 0.9, and a mean difference of 1.0.^15^ A *P*-value of < 0.05 was considered statistically significant for all comparisons. Data are presented as mean ± SD (text) or SEM (figures) with the 95% confidence interval (CI) for the mean difference. Data supporting the results are available in an electronic repository: https://doi.org/10.7910/DVN/MLJTCV.

## 3. Results

During the first cohort of rats tested, 4 rats assigned to group 2 were euthanized for reaching humane endpoints on the fifth day of the acute phase. Data (RGS, DAI, CBS) collected from these rats up to day 4 were included in the group 1 data set (except for necropsy data, which were not collected). The final sample sizes were unchanged because block randomization was maintained. Because of these animals reaching their humane endpoints, the remaining rats that had not yet been treated with DSS (group 1: n = 8, group 2: n = 10, controls: n = 10) received an acute phase that lasted 4 days, the water phase lasted 7 to 10 days, and the chronic phase lasted 3 days (group 2 rats displayed similar DAI scores as day 4 of the acute phase, average DAI 2/4). The burrowing data of one rat from the control group were excluded as it burrowed an average of 2 g during BL.

### 3.1. Disease Activity Index

**Figure 2.**
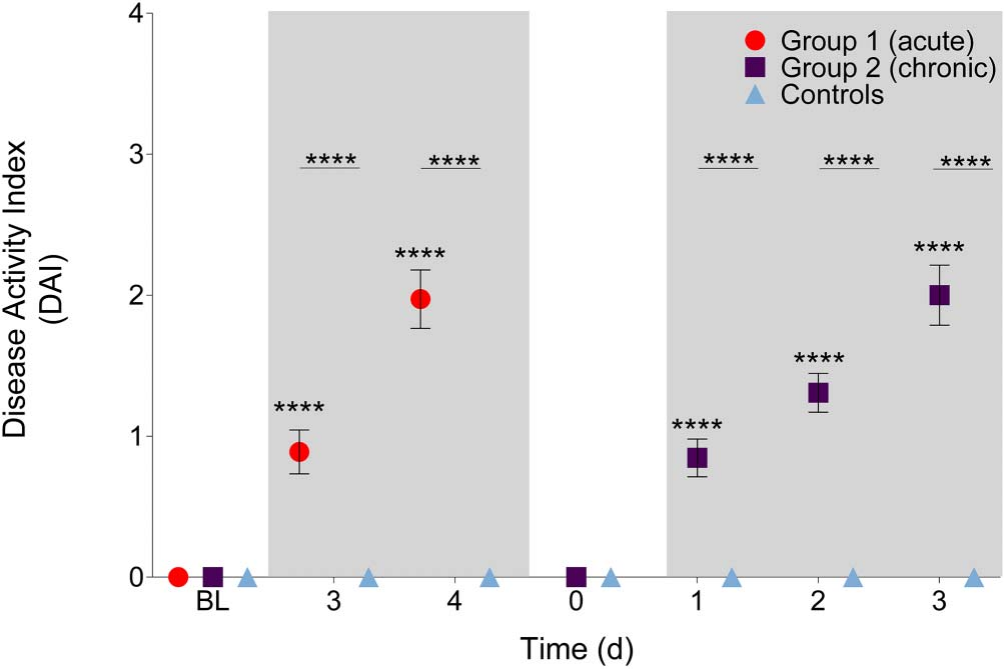
Disease Activity Index scores during the acute and chronic colitis phases. Disease Activity Index scores increased significantly during acute DSS exposure compared with BL and controls on days 3 and 4 (*P* < 0.0001). Disease Activity Index scores increased significantly during chronic DSS exposure compared with BL on day 0 (before DSS treatment began again) and controls on days 1, 2, and 3 (*P* < 0.0001). Shaded boxes represent when DSS treatment was given. ****P < 0.0001. Data presented as mean ± SEM. BL, baseline; DSS, dextran sulfate sodium.

During the acute phase, there were significant main effects for treatment and time (F (1, 23) = 95, *P* < 0.0001 and F (2, 46) = 59, *P* < 0.0001, respectively), and the interaction effect was significant (F (2, 46) = 59, *P* < 0.0001). Post hoc tests revealed that group 1 had increased DAI scores from BL and from the control group on days 3 (*P* < 0.0001, 95% CI [0.59–1.2]; *P* < 0.0001, 95% CI [01.2 to −0.54], respectively) and 4 (*P* < 0.0001, 95% CI [1.7–2.3]; *P* < 0.0001, 95% CI [−2.3 to −1.6], respectively; Fig. 2). During the chronic phase, there were significant main effects for treatment and time (F (1, 24) = 97, *P* < 0.0001 and F (4, 96) = 63, *P* < 0.0001, respectively), and the interaction effect was significant (F(4, 96) = 63, *P* < 0.0001). Post hoc tests revealed that the DAI scores of group 2 animals returned to the BL score of 0 before increasing significantly from BL and from controls on chronic phase days 1 (*P* < 0.0001, 95% CI [0.58–1.1]; *P* < 0.0001, 95% CI [−1.2 to −0.51] respectively), 2 (*P* < 0.0001, 95% CI [1.0–1.6]; *P* < 0.0001, 95% CI [−1.6 to −0.97] respectively), and 3 (*P* < 0.0001, 95% CI [1.7–2.3], *P* < 0.0001, 95% CI [−2.3 to −1.7] respectively). Animals from the control group maintained DAI scores of zero throughout.

### 3.2. Rat Grimace Scale

**Figure 3.**
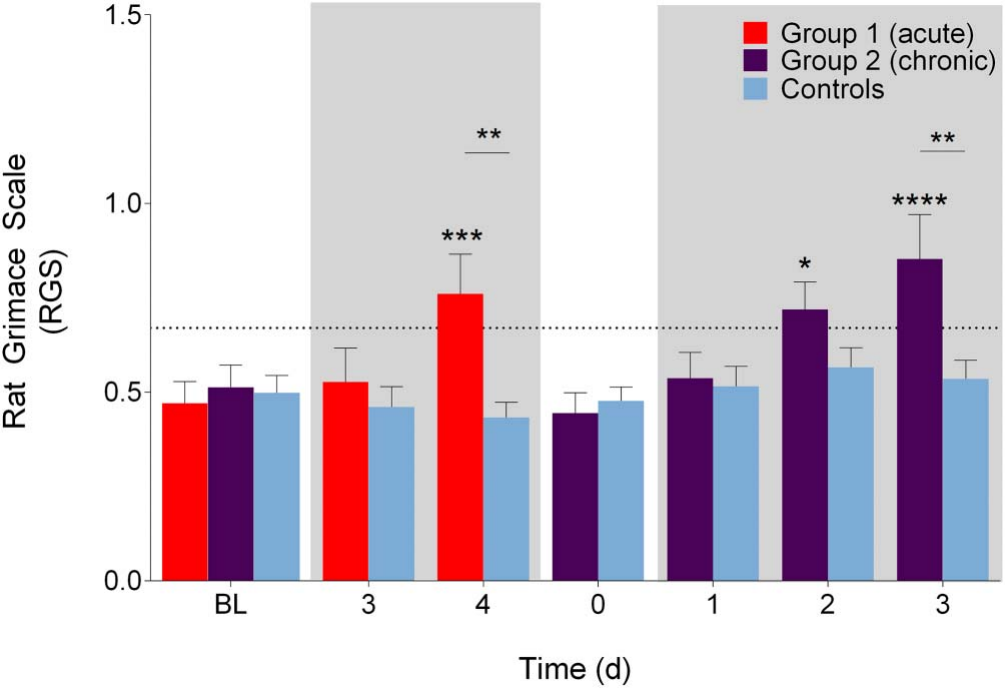
Rat Grimace Scale (video) scores during the acute and chronic phases. Significant increases from BL were observed on day 4 of the acute phase in group 1 and on days 2 and 3 of the chronic phase in group 2 (*P* < 0.05). Significant increases from controls were observed on day 4 during the acute phase and on days 2 and 3 during the chronic phase (*P* < 0.01). Broken horizontal line represents a derived analgesic intervention threshold.^24^ Shaded boxes represent DSS treatment phases. *P < 0.05. **P < 0.01. ***P < 0.001. ****P < 0.0001. Data presented as mean ± SEM. BL, baseline; DSS, dextran sulfate sodium.

With video scoring during the acute phase, there were significant main effects for treatment and time (F (1, 23) = 2.3, *P* = 0.14 and F (2, 46) = 3.6, *P* = 0.034, respectively) and a significant interaction effect (F (2, 46) = 7.8, *P* = 0.0012). Post-hoc tests revealed that group 1 showed increased RGS scores from BL (*P* = 0.0002, 95% CI [0.14–0.44]) and controls (*P* = 0.003, 95% CI [−0.56 to −0.09]) on day 4 (Fig. 3). During the chronic phase, there were significant main effects for treatment and time (F (1, 24) = 2.4, *P* = 0.14 and F (4, 96) = 6.8, *P* < 0.001, respectively) and a significant interaction effect (F (4, 96) = 3.6, *P* = 0.0092). Post hoc tests revealed that the RGS scores of group 2 decreased to their BL and control levels before increasing significantly from BL on chronic phase days 2 (*P* = 0.03, 95% CI [0.02–0.39]) and 3 (*P* < 0.0001, 95% CI [0.15–0.53]), crossing a previously established intervention threshold of 0.67.^24^ A significant increase compared with controls was visible on day 3 (*P* = 0.004, 95% CI [−0.56 to −0.08]).

Similar increases from BL and controls were observed from DSS-treated animals during the acute and chronic phases, when analyzed with real-time observations: there were significant main effects for treatment and time (F (1, 24) = 13, *P* = 0.0016 and F (4, 96) = 28, *P* < 0.0001, respectively) and a significant interaction effect (F (4, 96) = 16, *P* < 0.0001) (Suppl. Fig. 1A, available at http://links.lww.com/PR9/A40). The similarities between RGS real-time and video scores were also evident with a Bland–Altman of repeated measures; real-time scores had a bias of −0.11 when compared with video scores with limits of agreement ranging from −0.76 to −0.56 (Suppl. Fig. 1B, available at http://links.lww.com/PR9/A40).

### 3.3. Burrowing

**Figure 4.**
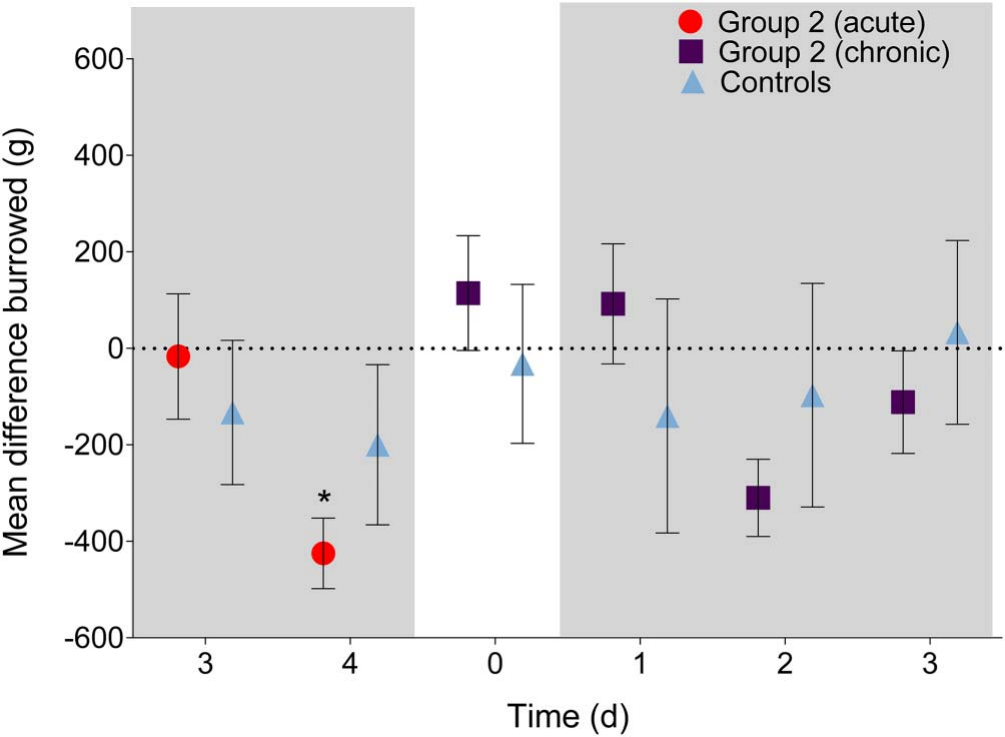
Mean difference in gravel displacement during acute and chronic colitis phases (shaded boxes). During both phases, no significant differences were observed between DSS treated and control rats (*P* > 0.99). A significant decrease from baseline was observed on day 4 (acute phase; *P* < 0.05). *P < 0.05. Data presented as mean ± SEM.

All rats burrowed to a similar degree at BL (group 2: 1404.2 ± 566.5 g; controls: 1330.0 ± 559.1 g). During the acute phase, there was a significant main effect of time (F (1, 23) = 5.9, *P* = 0.023) but not treatment (F (1, 23) = 0.12, *P* = 0.74) and a nonsignificant interaction effect (F (1, 23) = 3.0, *P* = 0.095). Post hoc tests revealed that there were no differences between the mean difference of gravel burrowed between group 2 and controls in both the acute and chronic phases (*P* > 0.99, all comparisons; Fig. 4). During the acute phase, group 2 rats burrowed significantly less than BL on day 4 (*P* = 0.03, 95% CI [36.2–813.7]). During the chronic phase, there were no significant differences observed (*P* > 0.05, all comparisons. 95% CI ranged from approximately −300 to 400).

### 3.4. Composite behavior score

**Figure 5.**
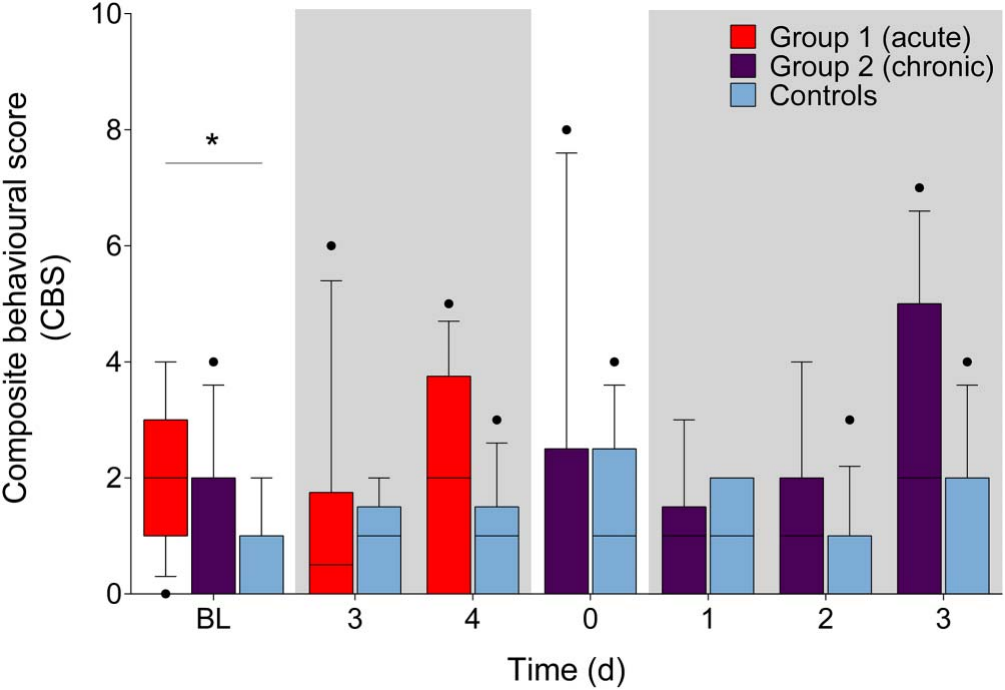
Summed frequency of 4 behaviours (back arch, stagger/fall, writhe, and twitch) evaluated during the acute phase and the chronic phase (shaded boxes). Differences between groups were identified at BL between group 1 and controls (*P* < 0.05). Differences within groups (from BL) were not observed. Shaded boxes represent when DSS treatment was given. Data presented as median (10–90 percentile). *P < 0.05. BL, baseline.

All behaviors except belly pressing were observed. During the acute phase, there was a significant main effect of treatment (F (1, 23) = 5.8, *P* = 0.024) but not time (F (2, 46) = 0.67, *P* = 0.52) and a non-significant interaction effect (F (2, 46) = 1.7, *P* = 0.20). During the chronic phase, there was a significant main effect of treatment (F (1, 24) = 5.6, *P* = 0.027) but not time (F (4, 96) = 1.7, *P* = 0.15) and a nonsignificant interaction (F (4, 96) = 0.79, *P* = 0.53). Post hoc tests revealed that the only difference was between group 1 and controls at BL (*P* = 0.02, 95% CI [−2.8 to −0.17], Fig. 5). No differences were observed between group 2 and controls or BL (Fig. 5, *P* > 0.05). Twitch frequency was the only behavior that identified treatment effects between group 2 and control rats during the third day of the chronic phase (*P* = 0.04, 95% CI [−2.7 to −0.021]; Suppl. Fig. 2D, available at http://links.lww.com/PR9/A40).

### 3.5. Microscopic score

After both acute and chronic phases, the microscopic score increased significantly from controls (*P* = 0.001, *P* < 0.0001, respectively; Table 4).

**Table 4:**
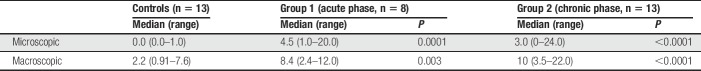
Microscopic and macroscopic scores of colon samples.

### 3.6. Macroscopic score

After both acute and chronic phases, significant increases from controls were evident (*P* = 0.003, *P* < 0.0001, respectively, Table 4).

## 4. Discussion

These results show the following: (1) Clinical signs of increasing disease severity (measured by the DAI) are reflected by an increase in RGS scores, but not by the CBS. (2) During acute colitis, as the DAI score increases, burrowing decreases. These data demonstrate that pain is likely to be present in DSS colitis models and increases concurrently with the presence of the clinical signs of the model (bleeding, loose stools, and weight loss). This is in line with previous studies showing that visceral nociception (assessed with a colorectal balloon pressure measurement) and referred hypersensitivity (assessed with the von Frey filaments) occurred.^13,30^ Previous work has described the temporal relationship between hypersensitivity and ongoing pain, showing that pain presents over a shorter time course than hypersensitivity (in a peripheral models of inflammation), a situation that may better model the human experience.^7,11^

The changes in RGS scores coincide with model severity as assessed with the DAI, confirming that pain is present when clinical signs of colitis are apparent. Furthermore, the mean RGS scores exceeded an established analgesic interventional threshold (0.67).^24^ This observation may be helpful in guiding manipulations in this model (decision to provide pain relief, response to treatment, and humane endpoints). The similar pattern of increase in RGS and DAI scores suggest that the DAI can be used as a proxy measure of pain. At the times when RGS scores crossed the analgesic intervention threshold, DAI scores were > 1, suggesting that this could be used as a proxy to trigger intervention. The successful application of facial expressions (Mouse Grimace Scale) has been previously applied to a murine colitis model (intrarectal allyl isothiocyanate), although no comparison was made with the DAI.^12^

With real-time RGS scoring, the same pattern of change upon exposure to DSS was also observed, providing further support for the notion that real-time RGS scoring is a useful and feasible method of rapid pain assessment.^18^ Furthermore, the closeness in RGS scores generated by real-time and standard scoring techniques supports the use of real-time scoring by a trained observer to routinely assess pain and welfare in this model. This means of rapid assessment could serve to identify humane endpoints or facilitate decisions regarding analgesia.

Unexpectedly, differences between DSS-treated and control rats were not identified with the CBS after DSS treatment. The behaviors evaluated (writhe, twitch, back arch, belly pressing, and fall/stagger) were previously validated in rats subjected to a laparotomy and were suggested as a potential tool to assess visceral pain.^26,29^ The incidence of some of these same behaviors has also been observed to increase in ureteral calculi and intestinal mucositis models.^10,33^ However, a slightly different combination of behaviors was observed in each model. For example, back-arching behavior was the only behavior observed in all 3 models (laparotomy, ureteral calculi, and intestinal mucositis models), whereas writhing was only observed after a laparotomy and intestinal mucositis model. This suggests that rats display a different combination of behaviors in different types of visceral pain models. Additional work is required to assess whether the addition of different behaviors will allow for discrimination between treatment groups in a DSS-colitis model.

Rats burrowed less on the same days where increases in DAI and RGS scores were observed during the acute phase (of group 2). This agrees with a previous mouse study that also observed reduced burrowing when mice were exposed to an acute dose of 2% DSS.^14^ However, this decrease was not sustained during the chronic phase, and no differences were observed compared with controls or BL. The absence of changes in burrowing behavior from BL during the chronic phase may reflect a lack of study power (reflected in wide 95% CI). Furthermore, the effect of chronic pain on burrowing behavior is currently unknown.

A limitation of this study is that a more comprehensive set of behaviors was not used as part of the CBS. Inclusion of additional behaviors may have better reflected the pain in this model. These behaviours could include abdominal licking and horizontal stretching, which were observed in mice following an allyl isothiocyanate–induced colitis model.^12^

In conclusion, the RGS was able to identify both acute and chronic phases of a colitis model, with changes occurring in tandem with clinical signs (reflected by the DAI). In addition, burrowing activity reflects ongoing acute visceral pain in this colitis model and may be changed in the presence of chronic pain. The concurrent changes observed in the DAI and RGS suggest that the DAI may be a proxy measure for pain that is simple to apply. Pain assessments with the real-time RGS or DAI are recommended to assess the efficacy of treatment or analgesics for colitis-related pain, to study visceral pain mechanisms or to ensure the well-being of rats with colitis.

## Disclosures

The authors declare no potential conflicts of interest with respect to the research, authorship, and/or publication of this article. Funding for this study was from the Discovery Grant from the Natural Sciences and Engineering Research Council of Canada (ID: 424022-2013, awarded to DSJP) and the American College or Laboratory Animal Medicine Foundation (awarded to DSJP). V.L. receives a stipend from the Fondation J-Louis Lévesque. Funders had no role in study design, data collection and analysis, or decision to publish.

## Supplementary Material

SUPPLEMENTARY MATERIAL

## Appendix A. Supplemental digital content

Supplemental digital content associated with this article can be found online at http://links.lww.com/PR9/A40.
