# Supplementary material for: Performance of behavioral assays: the Rat Grimace Scale, burrowing activity and a composite behavior score to identify visceral pain in an acute and chronic colitis model
Source: Pain Rep. 2019 Mar 5;4(2):e718. doi: 10.1097/PR9.0000000000000712 (PMC6455688; doi:10.1097/PR9.0000000000000712)
Supplement: SUPPLEMENTARY MATERIAL [file painreports-4-e718a-s001.docx]

**Supplemental Figure 1:** The Rat Grimace Scale scores (real-time observations) during the acute and chronic phases (A, shaded boxes) and B) a comparison between real-time and video scores with Bland-Altman analysis for repeated measures. A) Significant increases from baseline were evident on days 3 and 4 of the acute phase (p < 0.05) and on day 1, 2 and 3 during the chronic phase (p < 0.01). Significant increases from controls were evident on day 4 of the acute phase (p < 0.01) and on days 2 and 3 of the chronic phase. Horizontal dotted line represents a previously derived intervention threshold of 0.67 (Oliver et al., 2014). Data presented as mean ± SEM. B) Bias (-0.11, central broken horizontal line) reflects underestimation of video-based scores by real-time scores. Limits of agreement (broken horizontal lines) range from -0.76 to 0.56. *p < 0.05, **p < 0.01, ****p < 0.0001.





**Supplementary Figure 2:** Breakdown of the frequency of each behaviour assessed with the Composite Behaviour Score (CBS). Shaded boxes represent DSS treatment durations. Belly pressing was never observed. No significant differences were observed within groups (from baseline). A significant difference between groups (control and group 2) was only observed for twitch behaviour (p < 0.05). Data presented as median ± IQR.
